# Changing the paradigm: Elimination – Not only of cervical cancer

**DOI:** 10.1016/j.gore.2024.101445

**Published:** 2024-07-03

**Authors:** Jacob Bornstein, Koray Gorkem Sacinti, Mario Preti, Salem Billan, Hosna Razeghian, Colleen K. Stockdale

**Affiliations:** a The Research Institute of Galilee Medical Center, Galilee Medical Center, Nahariya, Israel; b Department of Obstetrics and Gynecology, Azrieli Faculty of Medicine of Bar-Ilan University, Nahariya, Israel; c Department of Obstetrics and Gynecology, Ankara University School of Medicine, Ankara, Turkey; d Division of Epidemiology, Department of Public Health, Hacettepe University Faculty of Medicine, Ankara, Turkey; e Department of Surgical Sciences, University of Torino, Torino, Italy; f Department of Oncology and Radiation, Head and Neck Center, Rambam Health Care Campus, Israel Institute of Technology, Haifa 31096, Israel; g Luigi Vanvitelli University of Campania School of Medicine and Surgery, Naples, Italy; h Department of Obstetrics & Gynecology, University of Iowa, Iowa City, Iowa, USA

**Keywords:** Human papillomavirus, HPV, Vulvar cancer, Anal cancer, Oropharyngeal cancer

## Abstract

The WHO’s initiative to eliminate cervical cancer by 2030 does not address the increasing incidence of vulvar, anal, and oropharyngeal cancers linked to high-risk HPV. Currently, the prevention of these three cancers faces various obstacles, such as a lack of specialized screening programs, well-defined management guidelines, and widespread public awareness. Without any interventions, the incidence of these three cancers will likely rise in the upcoming years, increasingly affecting younger individuals. We recommend expanding the WHO’s initiative to include vulvar, anal, and oropharyngeal cancers. This involves developing screening and management protocols similar to those for cervical cancer, implementing gender-neutral HPV vaccination programs, establishing clear referral pathways to specialized centers, promoting public awareness, and providing education to healthcare providers and high-risk individuals.

## Highlights

• Without interventions, incidences of vulvar, anal, and oropharyngeal cancers are expected to increase.
• Expanding the WHO’s cervical cancer initiative to cover vulvar, anal, and oropharyngeal cancers is essential.
• Reducing HPV-related cancer rates requires screening high-risk individuals, implementing gender-neutral HPV vaccinations, and enhancing awareness.

The global strategy of the World Health Organization (WHO) for eliminating cervical cancer by 2030 has united nations in a collective effort to eradicate this cancer, which is almost always associated with human papillomavirus (HPV) (WHO, 2023). However, there is a significant burden of other malignancies associated with high-risk HPV infection, such as vulvar, anal, and oropharyngeal cancers, which have not been included in the WHO’s elimination initiative. Currently, there is a lack of dedicated screening programs, comprehensive management protocols, and widespread public knowledge regarding these cancer types. If this situation remains unaddressed, it is estimated that the incidence of vulvar, anal, and oropharyngeal cancers will keep rising in the following years,

Vulvar cancer makes up roughly 4–6 % of gynecological malignancies, annually affecting 2–3 per 100,000 women (SEER Cancer Stat Facts: Vulvar Cancer, 2023). Vulvar squamous cell carcinoma (SCC) is the most common histological type of vulvar cancer, while melanoma, vulvar Paget disease, adenocarcinoma, and basal cell carcinoma are less frequent types. Vulvar SCC has traditionally been considered a disease of postmenopausal women, with over half of the affected patients being older than 70 years old (Schuurman et al., 2013). However, the incidence of vulvar SCC and its precancerous lesion, vulvar intraepithelial neoplasia (VIN), has significantly increased among younger women in recent decades. This increase is especially notable among women under the age of 60 in many high-income countries across Northern Europe, North America, and Australia, with vulvar cancer incidence increasing by 11.6 % every 5 years from 1988 to 2007 (Kang et al., 2017).

There are two distinct pathways for the development of vulvar SCC (Höhn et al., 2021). One pathway involves high-risk HPV types, most importantly HPV 16, 18, 31, and 33, where a vulvar high-grade squamous intraepithelial lesion (HSIL) acts as the precursor. This pathway accounts for the majority of vulvar precancerous lesions, with around 76–86 % HPV positivity in VIN cases (Faber et al., 2017, De Sanjosé et al., 2013). It primarily affects younger women and typically has a slow and infrequent progression rate. However, unlike cervical squamous cell carcinoma, which is almost exclusively linked to HPV, this HPV-related pathway gives rise to about 26–29 % of vulvar SCC cases (De Martel et al., 2017). The majority of vulvar SCC is attributed to the HPV-independent pathway that has differentiated-type vulvar intraepithelial lesion (dVIN) as a precursor and arises from chronic inflammatory dermatosis, like lichen sclerosus, in elderly, postmenopausal women (Vieira-Baptista et al., 2022). Therefore, when comparing these two precancerous lesion types, vulvar HSIL has a higher incidence, while dVIN has a higher malignant transformation.

Although both precancerous lesions of the vulva, vulvar HSIL and dVIN, typically present symptomatically with itching and pain, women often delay seeking medical attention. This delay might be due to embarrassment, stigma, misinterpretation of symptoms, lack of awareness, and healthcare access barriers (Rogers, 2021). Healthcare professionals may also hesitate to biopsy vulvar lesions since both pre-cancerous and cancerous lesions can resemble benign conditions like eczema. Consequently, vulvar cancers are frequently diagnosed at advanced stages, requiring extensive excisional treatments that result in disfigurement and decreased quality of life for these women. With the current absence of widely available screening tests and guidelines for early detection of vulvar precancerous lesions, it is critical for healthcare professionals to carefully examine the vulvar region whenever feasible, particularly during cytologic or HPV testing for cervical cancer screening (Preti et al., 2022). Equally important is to increase women’s awareness about the importance of vulvar self-examination to detect vulvar changes and seek treatment early on.

Not widely recognized is the fact that women with vulvar HSIL and SCC have an elevated risk of developing other HPV-related malignancies, such as anal SCC and its precursor lesion, anal HSIL, due to HPV field infection (Albuquerque et al., 2022). Almost entirely HPV-related, anal SCC has a worldwide incidence of 1–2 per 100,000 women. (de Martel et al., 2020, SEER Cancer Stat Facts: Anal Cancer, 2023) However, according to a recent *meta*-analysis, women with vulvar HSIL had an incidence ratio of anal cancer of 42 per 100,000 per year (95 % CI, 33–52), making them the third highest-risk group for anal cancer after human immunodeficiency virus (HIV)-positive men who have sex with men (MSM) and solid organ transplant recipient (SOTR) females who are ≥10 years post-transplantation (Clifford et al., 2021).

Several impediments hinder the prevention of anal HSIL and SCC in these high-risk women. They often lack awareness regarding their heightened susceptibility to developing additional concurrent or subsequent anogenital malignancies. Given the cessation of cervical cancer screening by the age of 64 and the unavailability of organized screening for anal cancer, these women miss out on essential routine medical examinations when they are most susceptible to developing anal HSIL and subsequently anal SCC. In addition, diagnosing anal HSIL and SCC can be challenging due to their non-specific presentations, including perianal pain, palpable mass, itchiness, and bleeding. This is especially true for primary care physicians, who may not have the necessary expertise to distinguish these malignancies from more commonly seen conditions like hemorrhoids. Furthermore, HPV infection is socially stigmatized similarly to other sexually transmitted diseases, making patients hesitant or ashamed to seek medical care at the onset of their symptoms, particularly when such an intimate area as the anogenital region is involved.

Research is ongoing to develop standardized, evidence-based screening and management guidelines for anal HSIL and SCC in these high-risk patients. The majority of the existing research has primarily centered around people living with HIV (PLWH) and MSM, as they are considered to have the highest risk. One noteworthy study is the Anal Cancer/HSIL Outcomes Research (ANCHOR) trial, (Lee et al., 2022) which compared anal cancer prevention strategies among PLWH with anal HSIL, revealing that treating anal HSIL, compared to active monitoring, substantially lowers the risk of anal SCC in this population. Further investigations are necessary to determine whether screening and treating HSIL in other high-risk populations, like patients with a history of genital HSIL, would provide similar benefits.

Several guidelines for anal cancer prevention have been established, but many were formulated before the ANCHOR study addressed uncertainties regarding the effectiveness of treatments for anal precancer. Most recently, the International Anal Neoplasia Society (IANS) has issued evidence-based guidelines for screening high-risk populations for anal cancer (Stier, 2024). Notably, these guidelines recommend starting screening within a year of diagnosing vulvar HSIL and SCC. Current screening strategies include anal cytology, high-risk HPV testing with genotyping for HPV16, and combined hrHPV-cytology testing, all of which have been shown to be effective. While these guidelines lay the groundwork for comprehensive screening programs, it remains crucial to collect longitudinal data on the risks and benefits of these screening methods to further refine the screening and management protocols for high-risk patients.

Another HPV-related malignancy that has seen a significant increase in incidence in the past decades is oropharyngeal cancer (OPC). In the USA, HPV-related OPC incidence increased by 225 % from 1988 to 2004, eventually surpassing the number of HPV-related cervical cancer cases (Chaturvedi et al., 2011, CDC, 2023). Cancers of the oropharyngeal region, like those affecting the base of the tongue, tonsils, soft palate, and uvula, have been historically attributed to alcohol and tobacco use. Nonetheless, studies suggest that around 70 % of OPCs are HPV-related, with HPV 16 being the most commonly detected type (Shinomiya and Nibu, 2023). The HPV status of OPC is regarded as an important factor in guiding therapy and predicting patient outcomes, as HPV positivity is associated with a better prognosis(Yakin et al., 2019). Male gender, white race, 40–59 age range, multiple sexual partners, and oro-genital and oro-anal sexual practices are other known risk factors (Gillison et al., 2008).

There are currently no screening methods and guidelines in place for the early detection of OPC, since the current technologies are inadequate for diagnosing precancer and early OPC and serum biomarkers are still under investigation (Lim and D'Silva, 2024).

However, healthcare professionals can play a crucial role in identifying cases at an early stage. They need to raise awareness about the potential signs and symptoms of OPC, encouraging patients to report any persistent symptoms that may indicate the condition. Given that lymph node metastasis occurs early on, primary care physicians should perform regular and thorough examinations of patients' head and neck areas to identify any lymph node enlargement. Dentists should also carefully examine the oropharynx during routine check-ups to look for any suspicious lesions that warrant further investigation (Tailor et al., 2020).

HPV vaccination, a crucial component of the WHO's strategy for cervical cancer elimination, is highly effective in reducing the incidence of HPV-related vulvar, vaginal, anal, oropharyngeal, and penile cancers as well. All available prophylactic HPV vaccines can provide protection against these cancers, with varying degrees of efficacy (Villa et al., 2020). Apart from the vaccine’s efficacy, vaccination success is also dependent on the proportion of the population vaccinated. For instance, the UK has one of the most successful national HPV vaccination programs in the world, achieving high coverage by offering free HPV vaccines to all 12- to 13-year-old schoolgirls since 2008 (Vaccine uptake guidance and the latest coverage data, 2023). As per a recent nationwide study involving a total of 13.7-million-year follow-up of females aged 20–30 years, this program has resulted in a remarkable 87 % reduction in cervical cancer incidence in the UK when compared to the unvaccinated cohort, almost eradicating the disease in women born after 1995 (Falcaro et al., 2021). The high coverage of such vaccination programs is expected to also provide protection to unvaccinated women through herd immunity.

The recommendation to vaccinate all genders against HPV (Gender Neutral Vaccination-GNV) has been shown to reduce the incidence of HPV-related cancers in males and enhance herd immunity. To improve gender-neutral HPV vaccination rates, implementing targeted strategies is crucial. The Healthy People 2030 initiative has set specific objectives to increase HPV vaccination rates among adolescents in the USA as part of its comprehensive roadmap for national health promotion and disease prevention (Healthy People 2030, 2024). It aims to ensure that 80 % of adolescents aged 13–15 get recommended doses of the HPV vaccine by 2030. To achieve this, the initiative recommends a multi-faceted approach that includes enhancing public awareness through campaigns, implementing school-based vaccination programs, sending patient reminders, advocating for insurance coverage and cost reduction, and improving the accessibility and convenience of vaccination services.

Observing the complete impact of HPV vaccination will require several years. In the meantime, the older generations who were not vaccinated and are vulnerable to HPV-related cancers can benefit from secondary preventive measures, such as early detection and timely treatment. Given the relatively low incidence of vulvar and anal cancers in the general population and the current limited availability of clinical knowledge, prioritizing the screening of high-risk patients, such as those diagnosed and treated for cervical intraepithelial neoplasia (CIN) 2/3, is essential (Kalliala et al., 2020). Furthermore, in order to increase the chance of detecting vulvar and anal precursor lesions, physicians should regularly examine the entire anogenital region of women with Pap or HPV test positivity.

We recognize that reducing the incidence and mortality of vulvar, anal, and OPC cancers demands significant logistical efforts. However, prevention of these three HPV-related neoplasias is still within reach. We call for the broadening of the WHO’s cervical cancer elimination initiative to encompass measures for eliminating vulvar, anal, and OPC cancers as well. These measures include launching gender-neutral HPV vaccination programs, refining screening and management algorithms similar to those used for cervical cancer for these three cancers, and establishing well-defined referral routes to specialized centers. Furthermore, it is essential to promote public awareness and provide comprehensive education to both healthcare providers and high-risk patients about these three cancers. Lastly, there is a critical need for performing standard clinical trials with innovative study designs to enhance screening protocols, early diagnosis, and treatment procedures.

**Source of Funding:** The authors received no financial support for the research, authorship, and/or publication of this article.

**Tweetable statement:** WHO's elimination initiative targets only cervical cancer, however, elimination of all HPV-related cancers is within reach.

## Declaration of Competing Interest

The authors declare that they have no known competing financial interests or personal relationships that could have appeared to influence the work reported in this paper.
